# Does Deworming Improve Growth and School Performance in Children?

**DOI:** 10.1371/journal.pntd.0000358

**Published:** 2009-01-27

**Authors:** David Taylor-Robinson, Ashley Jones, Paul Garner

**Affiliations:** 1 Division of Public Health, University of Liverpool, Liverpool, United Kingdom; 2 Centre for Medical Statistics and Health Evaluation, University of Liverpool, Liverpool, United Kingdom; 3 International Health Group, Liverpool School of Tropical Medicine, Liverpool, United Kingdom; *PLoS Neglected Tropical Diseases*, United States of America

## Background

The World Bank ranks soil-transmitted helminth infection as causing more ill health in children aged 5–15 years than any other infection. In light of this ranking, global agencies recommend regular, mass treatment with deworming drugs to children in developing countries [1],[2]. The World Health Organization (WHO) argues that “deworming helps meet the Millennium Development Goals” [3], in particular the six health-related goals: (1) eradicate extreme poverty and hunger; (2) achieve universal primary education; (3) promote gender equality and empower women; (4 and 5) reduce child mortality and improve maternal health; and (6) combat HIV/AIDS, malaria, and other diseases (http://www.un.org/millenniumgoals). However, deworming campaigns cost money to deliver, and so we must be clear that WHO statements about the impact of these programmes are based on reliable evidence.

In 2000, we systematically reviewed the reliable evidence from relevant controlled trials about the effects of anthelminth drugs for soil-transmitted helminth infection on child growth and cognition [4]. This systematic review, published in The Cochrane Database and the *BMJ* [4],[5], demonstrated uncertainty around the assumed benefit and concluded that it may be a potentially important intervention, but needed better evaluation.

The *BMJ* published a large number of letters that criticised the findings, including from authors at the World Bank, the WHO, the United States Centers for Disease Control and Prevention, and the Pan American Health Organization [6]. We do not feel that these criticisms were scientifically substantive enough to undermine the method or the conclusion (see “Authors' reply” in [6]). For example, several critics commented on the fact that the systematic review could not make any conclusions about the long-term effects of treatment—but, as we argued in our reply to these criticisms, “we were unable to find any randomised controlled trials that evaluated long term benefit, and the evidence of short term benefit was not, for us, convincing.” The research community quite correctly carried out further randomised controlled trials (RCTs) of repeated doses in community trials with longer follow-up compared with no intervention or placebo. In light of this additional research, we have now updated the original Cochrane review [7]. An author of one of the trials included in the 2000 review, Ed Cooper, criticised the review for not taking into account heterogeneity in parasite burdens [8]. Therefore, in the recently updated review, we conducted an additional subgroup analysis at trial level stratified by worm intensity and prevalence [7].

## Updated Cochrane Review

Our objectives were to summarize the effects of deworming drugs used to treat soil-transmitted intestinal worms on two outcomes: growth and school performance in children. The inclusion criteria were RCTs and quasi-randomised controlled trials that evaluated these outcomes. A total of 34 trials were included, including ten new trials since the 2000 review. Six trials in the 2000 review were excluded from the updated systematic review, as our methods of applying inclusion criteria have become more exacting [7]—for instance, trials with only two units of allocation, and trials that ignored randomisation in the analysis were excluded in the updated review.

Of the ten new trials, three were cluster randomised ([9],[10] and A. Hall, B. Nguyen, D. Bundy, D. Quan, S. Hong, et al. unpublished data). This design helps capture the potential positive impact of treating a whole community by reducing transmission. One unpublished trial from Vietnam, with 2 years follow-up, kindly provided by the authors, did not demonstrate a significant difference in weight gain. Clustering was not taken into account in the analysis, which artificially narrows the confidence intervals, but even with appropriate correction the result will remain non-significant (A. Hall, B. Nguyen, D. Bundy, D. Quan, S. Hong, et al. unpublished data). A second trial over 3 years, involving 27,995 children, also did not take clustering into account [9]. This second trial, published in the *BMJ*, reported a significant effect in weight gain in the intervention group; however, the children were randomised across 50 parishes, and when the authors kindly at our request adjusted the data for clustering, the data showed no significant difference between the intervention and control group (mean and 95% confidence interval of the difference in weight gain between the intervention and control group: 154 g, −19 g to 330 g) (H. Alderman, personal communication). The third trial, by Awasthi and colleagues, did correct for clustering, and no significant effect on weight gain was demonstrated in outcomes measured at 18 months of follow-up [10].

For trials where data were in a combinable form, the evidence has changed a little (Table S1). Weight gain after one dose of anthelminth drugs became just significant, and with confidence intervals that include potentially important weight gain values (Figure S1: WMD 0.34 kg, 95% CI 0.05 to 0.64). However, the effect was very different between studies, with large effects in small studies carried out prior to 1995 [7]. Whilst this effect was detected in trials in high prevalence areas, stratification of trials by worm intensity did not explain the heterogeneity, which was also present in many of the other analyses.

With multiple doses and longer term follow-up, no significant results were found in trials with data used in the meta-analysis for measures of changes in weight and height up to 1 year, and after 1 year [7]. However, an important cluster randomised trial in Zanzibar that adjusted appropriately demonstrated a difference in weight and height for multiple dosing within 1 year [11],[12].

Two trials reported school attendance, three reported development status, and four reported on cognition tests. Overall, five of the trials demonstrated no treatment effect, one trial noted an improvement in three out of ten cognitive tests used, and one trial did not report the results clearly.

## Implications of the Review

Deworming drugs are associated with increases in weight after a single dose. Generally, there remains no significant difference detected in multiple dose trials, apart from the cluster RCT in schools in Zanzibar [11],[12]; more recent cluster RCTs looking for an effect on weight gain have failed to demonstrate a difference. For school performance, data were very limited, and no convincing treatment effect was demonstrated. If benefit is not shown in RCTs, then it seems benefit is even less likely in “real world” operational conditions. A reasonable interpretation of the newly updated systematic review may be that deworming drugs used in targeted community programmes may be effective in relation to weight gain in the short term in some circumstances, but not in others; the potential long-term impact has not been demonstrated conclusively.

## Comment

With obvious relationships between worm infection, health status, and poverty, randomised trials help differentiate causality from confounding. Gulani and colleagues' systematic review of intestinal anthelmintic drugs on anaemia shows a marginal impact on haemoglobin, and the authors say that this could translate into a small effect on anaemia in populations where worms are common [13]. Our updated systematic review shows improvements in weight after one dose of deworming, but generally not in trials with longer follow-up, and no evidence of effect on school performance. Thus, there is a mismatch between the state of reliable, direct evidence of benefit and the benefit claimed by advocates of deworming.

One of the issues with current policies is that there seems to be conflation of the diseases in terms of claims of benefit. Deworming advocates describe the benefits of treating all helminths, including schistomiasis, filariasis, and soil-transmitted helminths [14]. Thus, evidence for the benefit of treating populations with schistosomiasis is fairly clear [15], as the infection has a very substantive effect on health. There is little debate that treatment helps people with this condition, but this does not mean a different drug treating a different helminth is equally effective.

Another problem in deworming trials arises when the results may be counfounded by concurrent use of deworming agents for multiple diseases. For example, one of the problems in interpreting a study by Miguel and colleagues, where albendazole was used in a quasi-randomised design, is that praziquantel was used in some but not other villages, with unknown confounding effects [16]. This study did not therefore meet the inclusion criteria in our updated systematic review, as discussed in [7].

We believe that evidence-informed policy needs to be underpinned by specific reviews that ensure that the interventions are effective in reducing morbidity or mortality for the conditions for which they are being given. We suggest that policy makers clarify that the research evidence has sometimes demonstrated benefits and sometimes has not. Guideline developers and policy makers at global, national, and local levels should be allowed to consider the evidence carefully before committing to investing existing resources in delivering these programmes. Our interpretation of the data is that deworming policies applied to whole populations may possibly have benefits in some circumstances, but not in others. The debate remains open, but the large DEVTA trial (deworming and vitamin A; http://www.ctsu.ox.ac.uk/projects/devta), the world's largest ever RCT, should clarify if deworming is worthwhile. The trial includes over a million children randomised in a cluster design with mortality as the primary outcome [17].

## Supporting Information

Figure S1 Forrest plot of trials measuring change in weight after one dose of deworming, grouped by worm prevalence and intensity
(1.33 MB TIF)

Table S1 Change in nutritional status in trials in children comparing anthelminth drugs to no specific anthelminth treatment: reported after single dose and multiple doses
(0.05 MB DOC)
