# Supplementary figures and images for: Does Deworming Improve Growth and School Performance in Children?
Source: PLoS Negl Trop Dis. 2009 Jan 27;3(1):e358. doi: 10.1371/journal.pntd.0000358 (PMC2627941; doi:10.1371/journal.pntd.0000358)

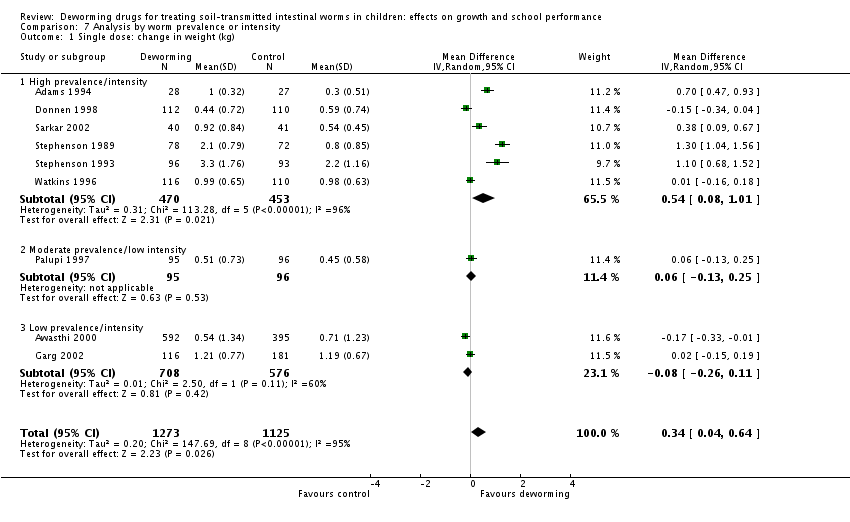

Supplement: Figure S1 — Forrest plot of trials measuring change in weight after one dose of deworming, grouped by worm prevalence and intensity (1.33 MB TIF) [file pntd.0000358.s001.tif]
