# Supplementary material for: Does Deworming Improve Growth and School Performance in Children?
Source: PLoS Negl Trop Dis. 2009 Jan 27;3(1):e358. doi: 10.1371/journal.pntd.0000358 (PMC2627941; doi:10.1371/journal.pntd.0000358)
Supplement: Table S1 — Change in nutritional status in trials in children comparing anthelminth drugs to no specific anthelminth treatment: reported after single dose and multiple doses (0.05 MB DOC) [file pntd.0000358.s002.doc]

**Table S1. Change in nutritional status in trials in children comparing anthelminth drugs to no specific anthelminth treatment: reported after single dose and multiple doses**

| **2000 results** | | | | | **2007 results** | | |
| --- | --- | --- | --- | --- | --- | --- | --- |
|  | Outcomes | #Trials | #Randomised | WMD (95% CI) | #Trial | #Randomised | WMD (95% CI) |
|  |  |  |  | Random effects model |  |  | Random effects model |
| **Single dose (outcomes <1 year)** | Weight (kg): | 7 | 2007 | 0.38 ( -0.01 to 0.77) ^ | 9 | 2448 | 0.34 (0.05 to 0.64) ^ |
|  | Height (cm): | 7 | 2008 | 0.09 ( -0.13 to 0.31) ^ | 9 | 2449 | 0.04 (-0.16 to 0.23) |
|  | MUAC (cm): | 5 | 823 | 0.23 ( -0.03 to 0.48) ^ | 5 | 823 | 0.23 [-0.03, 0.48] ^ |
|  | Triceps skinfold (mm): | 3 | 394 | 1.27 ( 0.74 to 1.80) ^ | 3 | 394 | 1.27(0.74 to 1.80) ^ |
|  | Subscapular skinfold (mm): | 3 | 394 | 1.18 ( 0.90 to 1.46) | 3 | 394 | 1.18 (0.90 to 1.46) |
| **Multiple dose trials (outcomes < year)** | Weight (kg): | 10 | 6107 | 0.15 ( 0.0 to 0.30) | 6 | 1714 | 0.05 (-0.24 to 0.33) ^ |
|  | Height (cm): | 10 | 6108 | 0.06 ( -0.07 to 0.19) | 6 | 1715 | -0.02 (-0.15 to 0.12) |
|  | MUAC (cm): | 3 | 593 | 0.07 ( -0.28 to 0.42) ^ | 4 | 658 | 0.06 (-0.24 to 0.36) ^ |
|  | Triceps skinfold (mm): | 1 | 188 | 1.80 (1.52 to 2.08) | 2 | 254 | 1.48 (1.21 to 1.74) ^ |
|  | Subscapular skinfold (mm): | 1 | 188 | 1.50 (1.23 to 1.77) | 1 | 188 | 1.50 (1.23 to 1.77) |
| **Multiple dose trials (outcomes > year)** | Weight (kg): | 2 | 1089 | 0.43 ( -0.61 to 1.47) ^ | 3 | 1219 | 0.46 (-0.47 to 1.39) ^ |
|  | Height (cm): | 2 | 1089 | -0.22 ( -0.84 to 0.40) fixed effect | 3 | 1219 | -0.26 (-0.84 to 0.31) fixed effect |

^I^2^ test for heterogeneity > 75%
